# Effects of CuZnAl Particles on Properties and Microstructure of Sn-58Bi Solder

**DOI:** 10.3390/ma10050558

**Published:** 2017-05-19

**Authors:** Fan Yang, Liang Zhang, Zhi-quan Liu, Su Juan Zhong, Jia Ma, Li Bao

**Affiliations:** 1 School of Mechanical and Electrical Engineering, Jiangsu Normal University, Xuzhou 221116, China; yangfan291233@126.com; 2 Institute of Metal Research, Chinese Academy of Sciences, Shenyang 110016, China; zqliu@imr.ac.cn; 3 State Key Laboratory of Advanced Brazing Filler Metals & Technology, Zhengzhou Institute of Mechanical Engineering, Zhengzhou 450001, China; zhongsujuan067211@126.com (S.J.Z.); majia052912@sina.com (J.M.); baoli064832@sina.com (L.B.)

**Keywords:** Sn-58Bi solder, lead-free solder, microstructure

## Abstract

With the purpose of improving the properties of the Sn-58Bi lead-free solder, micro-CuZnAl particles ranging from 0 to 0.4 wt % were added into the low temperature eutectic Sn-58Bi lead-free solder. After the experimental testing of micro-CuZnAl particles on the properties and microstructure of the Sn-58Bi solders, it was found that the wettability of the Sn-58Bi solders was obviously improved with addition of CuZnAl particles. When the addition of CuZnAl particles was 0.2 wt %, the wettability of the Sn-58Bi solder performed best. At the same time, excessive addition of CuZnAl particles led to poor wettability. However, the results showed that CuZnAl particles changed the melting point of the Sn-58Bi solder slightly. The microstructure of the Sn-58Bi solder was refined by adding CuZnAl particles. When the content of CuZnAl addition was between 0.1 and 0.2 wt %, the refinement was great. In addition, the interfacial IMC layer between new composite solder and Cu substrate was thinner than that between the Sn-58Bi solder and Cu substrate.

## 1. Introduction

As a kind of conventional solders, Sn–Pb solder was widely used in microelectronic packaging for past decades. However, with the awareness of environmental protection raised, many countries and international organizations have limited or banned the use and production of Sn–Pb solder by a series of legislations due to its being harm to the environment and human health [1,2,3]. For example, the European Union promulgated waste electronic and electrical equipment (WEEE) and the restriction of hazardous substances (RoHS) [4]. Thus lead-free solders raised highest interesting of many researchers and it is important for the world to develop a series of new lead-free solders.

Nowadays, many researchers focus on Sn based solders including Sn–Zn, Sn–Cu, Sn–Ag–Cu, and Sn–Bi [5]. Among these solders, the wettability of the Sn–Zn solder is not good because of the existence of Zn and the resistance to oxidation of the Sn–Zn solder is worse than others. Eutectic Sn–Cu solder has high melting point of 227 °C. It is higher than that of Sn–Pb solder. Moreover, its wettability and mechanical properties are inferior to other Sn based solders [6]. Sn–Ag–Cu lead-free solder was deemed as the most promising lead-free solder as a substitute for the traditional Sn–Pb solder on account of its properties which are close to that of Sn–Pb solder [7]. However, the Sn–Ag–Cu solder also has a high melting point and its wettability is poor. During the service period, the intermetallic compounds layer increases rapidly, resulting in the decline of reliability owing to the large amount of Sn. In addition, the cost of the Sn–Ag–Cu solder is higher than other solders because it contains Ag element. The eutectic Sn-58Bi solder has low melting point of 139 °C, good mechanical properties, effective creep resistance, and higher reliability [8,9]. However, the Bi brittle behavior is obvious in Sn-58Bi solder and it occupies a large proportion which degrades the tensile strength and elongation and it results in a negative impact on the solder joint. Meanwhile, the coarsening rate of Bi is higher and the segregation of Bi phase in Sn-58Bi solder is serious [10]. Therefore, many researchers selected alloy elements, rare earth, nano-sized, and micro-sized particles to improve the properties of Sn-58Bi solder and refine its microstructure.

In recent years, many researchers have added additives into the Sn-58Bi solder and found that the properties of the Sn-58Bi solder were strengthened. For example, Miao et al. [11] found that adding Cu element into the Sn-58Bi solder decreased the coarsening rate of Bi-rich phase under thermal aging at 120 °C. The addition of 0.7% Zn not only suppressed the coarsening of Bi phase but also the growth of Cu–Sn intermetallic compounds during liquid-state aging [12]. Ma et al. [13] found that the addition of Zn element into the Sn-58Bi solder could suppress the coarsening of Bi and the growth of IMCs between the Sn-58Bi solder and Cu substrate. Additionally, it also increased the tensile strengths of Sn-58Bi solder and improved the creep behavior remarkably. A study of microstructure and mechanical properties of the Sn-58Bi solder with 0.5% In addition has been done [14]. It was found that the tensile strength reduced, but the shear strength improved due to the suppression of the growth of IMCs and Bi coarsening within the solder bulk. Shiue et al. [15] added rare earth element La into the Sn-58Bi solder and found that the addition of 0.5% La reduced the eutectic point slightly but inhibited the growth of the IMC layers. Shin et al. [16] proposed that the addition of SiC nano-particles enhanced the shear strength of the Sn-58Bi solder by 6% and had thinner eutectic structures and grain sizes, but it did not affect the melting temperature. Adding Y_2_O_3_ particles (3–5 µm) improved the wettability of Sn-58Bi solder [17]. In addition, the wettability was about increased by 20% when the percentage of Y_2_O_3_ particles was 1.0%. In contrast, Liu et al. [18] assumed that the wettability of Sn-58Bi solder with 0.1% Cu_6_Sn_5_ particles got worse and the size of the interfacial IMC got lager. Nevertheless, the shear strength of the Sn-58Bi solder was strengthened.

In this paper, the effects of micro-CuZnAl particles addition on wettability, melting temperature, microstructure of the Sn-58Bi solder, and the IMC layer between the composite solder and Cu substrate were investigated.

## 2. Experimental Procedures

Sn-58Bi solder bearing different weight percentages of micro-CuZnAl particles addition ranging from 0 to 0.4% were prepared by stirring mechanically for 30 min to homogenize the solder alloys. The investigated composite solder alloys were shown in Table 1.

**Figure 1:**
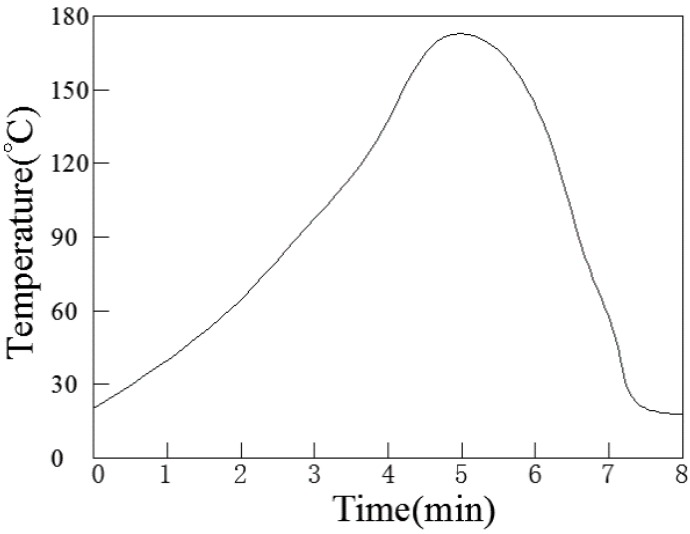
Reflow soldering curve of Sn–Bi base solders.

**Figure 2:**
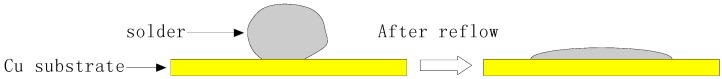
Schematic illustration of Sn–Bi base solders’ spreading test.

First, the wettability is an important property for solders. The wettability of Sn-58Bi-*x*CuZnAl can be analyzed by spreading area testing. The geometric dimension of Cu substrates used in this test was 25 × 25 × 0.1 mm^3^. They were degreased with ethanol (with 1 vol % HCl) to remove their surface oxides and contaminants. In the test, the weight of solder sphere is 0.2 g ± 0.001 g for testing. Then put them into the reflow oven to conduct reflow soldering. The reflow soldering curve was implied in Figure 1. After reflowing and cooled to the room temperature in the air, samples shaped well were selected to be photographed and transferred to the computer. The spreading area of samples was calculated by the Image-J software. The schematic drawing of the reflow soldering was indicated in the Figure 2.

The melting characteristic of Sn-58Bi-*x*CuZnAl solder alloys were measured by a different scanning calorimetric (DSC) with 15 mg of composite solder alloys was put in Al_2_O_3_ crucible and it was scanned from 25 to 250 °C at a rate of 10 °C/min.

The microstructure of each type of composite solders and the intermetallic compounds layers was observed by using scanning electron microscopy (SEM) with a voltage of 15 keV. Cross-sections of the solder/Cu interfaces mounted in resin, sanded with sandpapers, polished with 0.25 µm diamond paste, and etched with 95% CH_3_OH and 5% HNO_3_ solution for 1~2 s.

## 3. Results and Discussion

### 3.1. CuZnAl Particles

**Figure 3:**
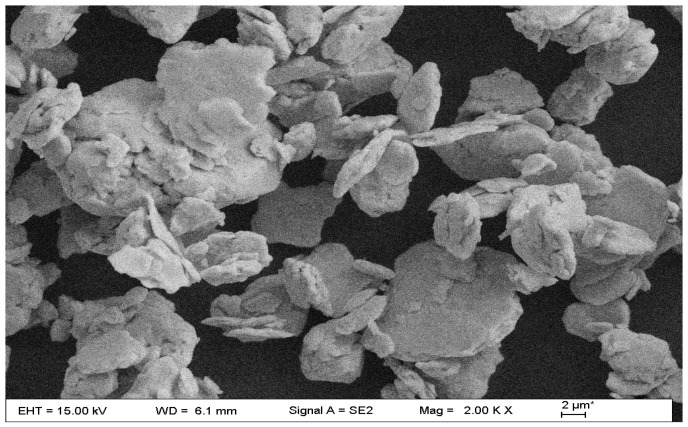
SEM of CuZnAl particles.

The Figure 3 showed the SEM picture of micro-CuZnAl particles which were added into the Sn-58Bi solder in this research. In the picture, the shapes of CuZnAl particles are plate-shaped and the size ranges from 0.5 µm to about 6 µm. In addition, there is agglomeration in these particles.

### 3.2. Wettability

**Figure 4:**
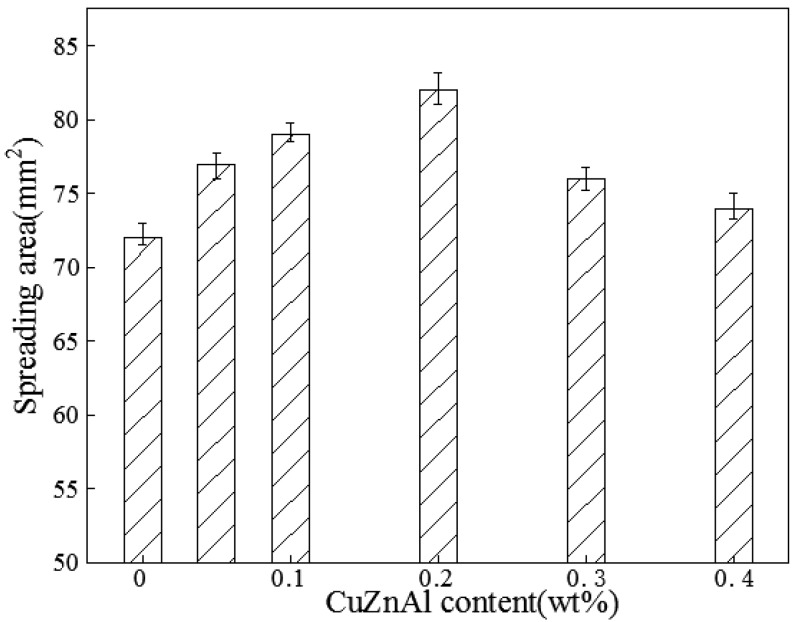
Spreading area of Sn-58Bi-*x*CuZnAl composite solders.

Wettability which plays an important role in the good bonding between the solder alloys and substrates is one of the fundamental properties of the solder alloys [19]. It is also a key role to reliability of electronic products [20]. Generally, the larger the area, the better the wettability. The results of spreading areas of Sn-58Bi-*x*CuZnAl composite solders were revealed in Figure 4. It was found that the spreading area was increased and then decreased with the increasing of CuZnAl particles contents. When the content was 0.2 wt %, the spreading area was highest. Compared with Sn-58Bi solder, the spreading area of the Sn-58Bi solder with 0.2% CuZnAl particles increased by 13.9% from 72 to 82 mm^2^, which means that in these solder alloys the wettability of Sn-58Bi with 0.2 wt % CuZnAl particles was optimal. Furthermore, the wettability performed worse when the addition of CuZnAl was exceeded. Surface segregation was possible because particles were active. They could reduce the surface tension. At the same time, they also improved the fluidity of solder during reflow soldering. However, when the CuZnAl particles addition was further increased, it could inhibit the wettability because of the particles’ agglomeration. As the Figure 4 shown, the spreading area was decreased when the content of CuZnAl particles exceeded 0.2 wt %. However, their wettability was also better than that of the Sn-58Bi solder.

### 3.3. Melting Characteristic

**Figure 5:**
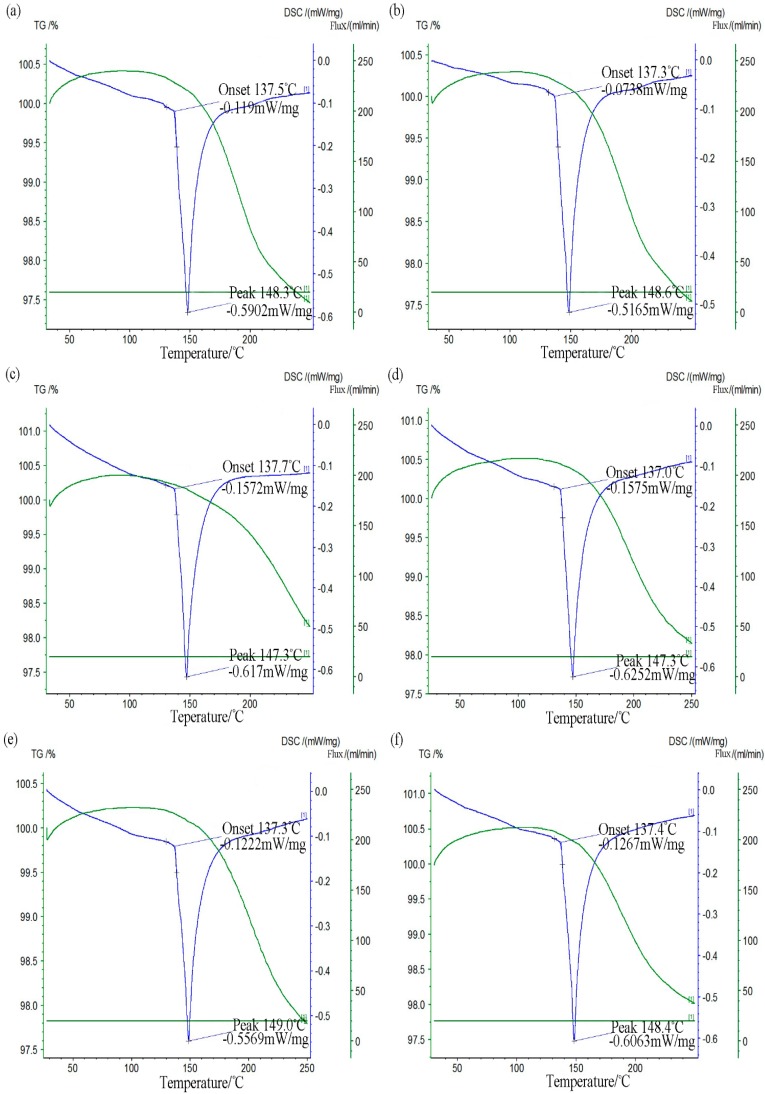
The melting characteristics of Sn-58Bi-*x*CuZnAl; (**a**) *x* = 0; (**b**) *x* = 0.05; (**c**) *x* = 0.1; (**d**) *x* = 0.2; (**e**) *x* = 0.3; (**f**) *x* = 0.4.

The melting characteristic also plays an important role in reflow soldering. The low temperature Sn-58Bi lead-free solder has lower melting point of 139 °C. It is necessary to study the effect of addition of CuZnAl micro-particles on the melting temperature of the Sn-58Bi solder. The DSC curves of Sn-58Bi-*x*CuZnAl solders were displayed in Figure 5. As can be seen from the pictures, it revealed that the addition of CuZnAl particles influenced the melting temperature of the Sn-58Bi solder slightly. The onset temperature ranged from 137.0 to 137.7 °C. So their solidus temperatures was close to each other. At the same time, their liquidus temperature varied from 147.3 to 149.0 °C and the variation was not large. Thus, the melting characteristic of the Sn-58Bi solder with CuZnAl particles did not change remarkably. It was due to minor addition of CuZnAl particles. The current equipment also could meet the requirement of the reflow soldering.

### 3.4. Microstructure of Solder Bulk

**Figure 6:**
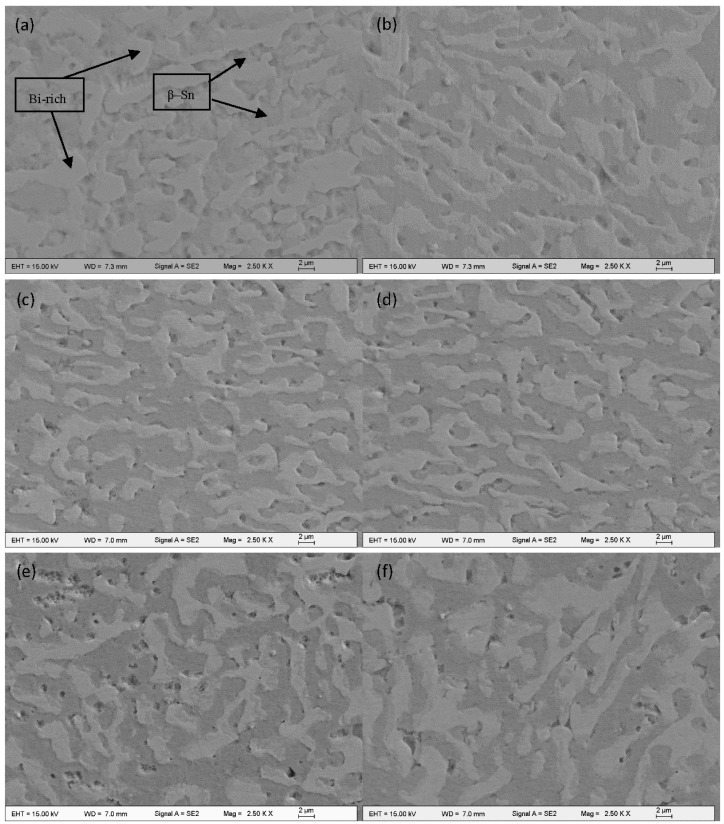
Microstructures of Sn-58Bi-*x*CuZnAl after reflow; (**a**) *x* = 0; (**b**) *x* = 0.05; (**c**) *x* = 0.1; (**d**) *x* = 0.2; (**e**) *x* = 0.3; (**f**) *x* = 0.4.

The microstructure of the eutectic Sn-58Bi solder is made up of typical grey β–Sn phase and white Bi-rich phase showed clearly in Figure 6a. The microstructures of Sn-58Bi solder bearing different content of CuZnAl particles addition ranging from 0 to 0.4 wt % were revealed in Figure 6. As can be seen from Figure 6, the changes of the microstructures of composite solders were obvious. At first, the shape of Sn-58Bi was classic lamellar. When the CuZnAl particles were added into Sn-58Bi solder, microstructure of these composite solders was refined. It was found that the content of CuZnAl addition was 0.1% or 0.2%, the coarsening of Bi phase was reduced greatly. However, with CuZnAl particles further added, microstructure gradually became coarser. Furhtermore, the microstructure was coarser than that of Sn-58Bi older when the content of CuZnAl addition was 0.4%. The reason why it happened this phenomenon was due to agglomeration of particles resulting in bad fluxility with further CuZnAl addition. When the content of CuZnAl particles addition was minor, CuZnAl particles diffused uniformly in Sn-58Bi solder. However, the agglomeration of CuZnAl particles became worse with the content increased. The refining effect on microstructure of Sn-58Bi solder weakened. So the optimal content of the greatest refinement was between 0.1% and 0.2%.

### 3.5. Interfacial IMC Layer

**Figure 7:**
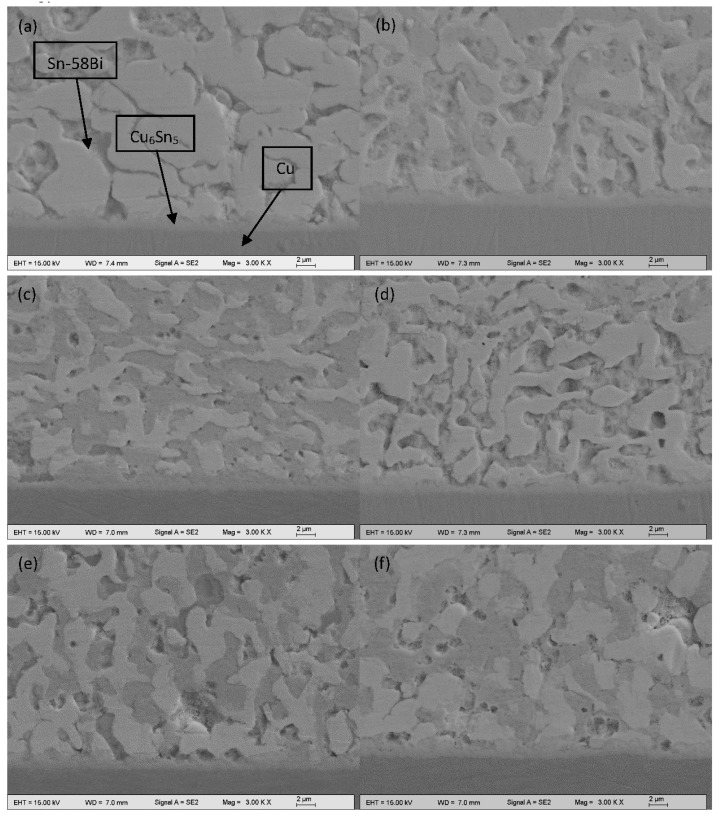
Interfacial IMC layers of Sn-58Bi-*x*CuZnAl after reflow; (**a**) *x* = 0; (**b**) *x* = 0.05; (**c**) *x* = 0.1; (**d**) *x* = 0.2; (**e**) *x* = 0.3; (**f**) *x* = 0.4.

**Figure 8:**
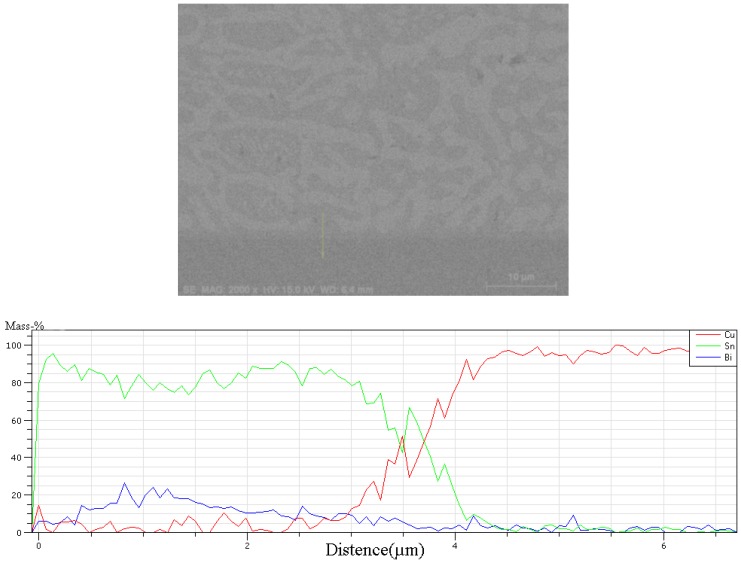
SEM morphology and EDX line scan profiles of interface between Sn-58Bi and Cu substrate.

**Figure 9:**
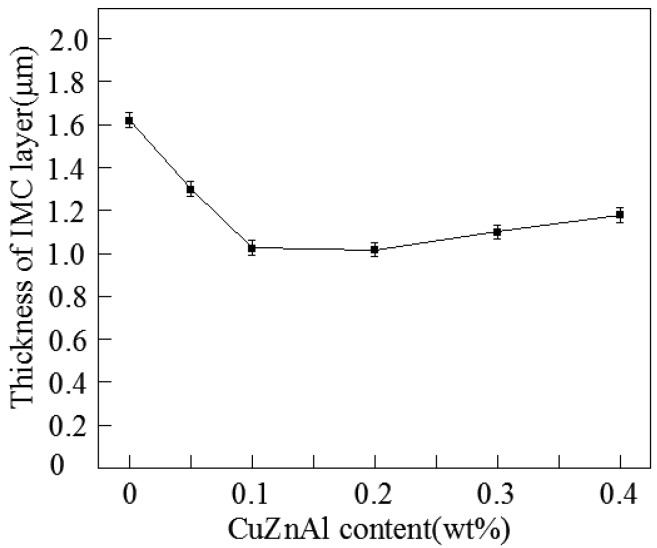
Average intermetallic compound layer thickness of Sn58Bi-xCuZnAl solder joints.

The interfacial IMC layers between Sn-58Bi-*x*CuZnAl composite solder and Cu substrate after the reflow were showed in the Figure 7. In the Figure 8, at the same time, Cu_6_Sn_5_ phase was detected by EDX line scan profiles analysis. The average thickness of IMC layers (χ) was measured by the total area of the IMC layer divided by the length of the IMC layer, as described by the following equation [21]:

(1) $$\chi =\;\frac{A}{L}$$

where *A* is the integral area of IMC layer; L is the length of the IMC layer. As could be seen from the Figure 9, when the CuZnAl particles were added into the Sn-58Bi solder, the thickness of the interfacial IMC layers was reduced obviously. In the Figure 7a, the shape of interfacial IMC layers was scallop without CuZnAl particles. The thickness of the IMC layer was 1.62 µm. Nevertheless the thickness of the interfacial IMC layer decreased from 1.62 to 1.28 µm after 0.05 wt % CuZnAl particles were added into the Sn-58Bi solder. Furthermore, the trend of abatement continued with further CuZnAl particles addition. When the addition of CuZnAl particles was 0.2%, the thickness of IMC layer decreased to 1.03 µm. The interface became flatter. However, as the content of CuZnAl particles increased, the thickness of IMC layers between the composite solders and Cu substrate became slightly thicker. Because CuZnAl particles diffused in the Sn-58Bi solder and the interface between the composite solder and Cu substrate. It reduced the grain boundary migration activation energy.

## 4. Conclusions

In this paper, the effects of CuZnAl micro-particle addition on the wettability, melting temperature, microstructural evolution, and intermetallic compounds layer of Sn-58Bi solder were investigated. The addition of CuZnAl particles improves the wettability of the Sn-58Bi solder and the optimum content is 0.2%. However, CuZnAl particles do not affect the melting point of the Sn-58Bi solder significantly. The CuZnAl particles refine the microstructure and suppress the growth of the IMC layer between the solder and Cu substrate.

## Figures and Tables

**Table 1:** Series of investigation solders.

Sample Number	1	2	3	4	5	6
CuZnAl/(wt %)	0	0.05	0.1	0.2	0.3	0.4
Initial alloy	Sn-58Bi					
